# Equity in long-lasting insecticidal nets and indoor residual spraying for malaria prevention in a rural South Central Ethiopia

**DOI:** 10.1186/s12936-016-1425-0

**Published:** 2016-07-16

**Authors:** Alemayehu Hailu, Bernt Lindtjørn, Wakgari Deressa, Taye Gari, Eskindir Loha, Bjarne Robberstad

**Affiliations:** Center for International Health, University of Bergen, Bergen, Norway; Department of Reproductive Health and Health Service Management, School of Public Health, Addis Ababa University, P.O. Box 9086, Addis Ababa, Ethiopia; Department of Preventive Medicine, School of Public Health, Addis Ababa University, Addis Ababa, Ethiopia; School of Public and Environmental Health, Hawassa University, Hawassa, Ethiopia; Center for Intervention Science in Maternal and Child Health (CISMAC), University of Bergen, Bergen, Norway

**Keywords:** Ethiopia, Equity, Malaria prevention, LLIN, IRS, Inequality analysis, Concentration index

## Abstract

**Background:**

While recognizing the recent achievement in the global fight against malaria, the disease remains a challenge to health systems in low-income countries. Beyond widespread consensuses about prioritizing malaria prevention, little is known about the prevailing status of long-lasting insecticidal nets (LLINs) and indoor residual spraying (IRS) across different levels of wealth strata. The aim of this study was to evaluate the socioeconomic related dimension of inequalities in malaria prevention interventions.

**Methods:**

This study was conducted in July–August 2014 in Adami Tullu district in the South-central Ethiopia, among 6069 households. A cross-sectional data were collected on household characteristics, LLIN ownership and IRS coverage. Principal component analysis technique was used for ranking households based on socioeconomic position. The inequality was measured using concentration indices and concentration curve. Decomposition method was employed in order to quantify the percentage contribution of each socioeconomic related variable on the overall inequality.

**Results:**

The proportion of households with at least one LLIN was 11.6 % and IRS coverage was 72.5 %. The Erreygers normalized concentration index was 0.0627 for LLIN and 0.0383 for IRS. Inequality in LLIN ownership was mainly associated with difference in housing situation, household size and access to mass-media and telecommunication service.

**Conclusion:**

Coverage of LLIN was low and significant more likely to be owned by the rich households, whereas houses were sprayed equitably. The current mass free distribution of LLINs should be followed by periodic refill based on continuous monitoring data.

## Background

In the last decade, the global fight against malaria reaches on promising phase. Between 2000 and 2013, malaria mortality was reduced by 47 % worldwide and by 54 % in Africa. During the same period, deaths from malaria dropped by half in Ethiopia. However, malaria still remains to be one of the major challenges for the health system in low-income countries. The disease is widespread around the globe, putting approximately 3.3 billion people at risk [1].

Malaria is one of the leading health problems in Ethiopia. Records from the Ministry of Health (MoH) reveal that more than 75 % of the total land mass is endemic and about 68 % of the population is living in a malarious area [2]. The World Health Organization (WHO) report more than 3.7 million cases of malaria infection for the year 2012 [3], and more than 2.1 million of cases for 2013 [4], in Ethiopia. Malaria is also one of the leading causes of outpatient visits, inpatient admissions and hospital deaths. In the malaria endemic districts of Oromia region, malaria account for up to 29 % of all outpatient visits [5], while in Adami Tullu district, where this paper emanates, malaria parasitic prevalence peaks up to 10.4 % [6]. A recent study by Gari et al. similarly reported a higher incidence of malaria cases (4.6 cases per 10,000 person-weeks of observation) from the same area [7].

The incidence peaks biannually from September to December and April to May, both coinciding with harvesting seasons [8]. This has a serious consequence for Ethiopian farmers whom constitute the vast majority of the total population. The consequences regard both the farmers, who are dependent on subsistence agriculture for livelihood, but also more broadly the economic development of the country. Studies consistently show also malaria imposes heavy sanctions on economic growth and causes household impoverishment [9, 10].

Malaria causes multifaceted problems which demand priority as well as synergistic intervention. Prevention of malaria using long-lasting insecticidal nets (LLINs) and indoor residual spraying (IRS) has been demonstrated to be cost-effective interventions in different contexts. A systematic review indicates a median incremental cost effectiveness ratio (ICER) per disability adjusted life year (DALY) averted of $27 for insecticide-treated nets (ITNs), and $143 for IRS [11]. These tools have been scaled up in the last decade aiming towards a universal access and to interrupt malaria transmission in malaria-endemic developing countries [12].

The results of the last two malaria indicator surveys (MIS) showed a remarkable stride in malaria prevention and control services in Ethiopia. For example: ITN ownership in malaria endemic areas improved from 3.4 % in 2005 [13] to 65.6 % in 2007 [14]. Overall, 68 % of households in malaria endemic areas were protected by at least one LLIN or indoor residual spraying of households with insecticide [15]. Thirty percent of IRS targeted areas were sprayed in 2007 and in 2008 the coverage increased to 50 % [16]. So far, (since 2005 till 2014), a total of 64.2 million ITNs have been distributed [17]. Currently, Ethiopia aims to achieve universal coverage by distributing one LLIN per 1.8 persons through mass and free distribution campaigns at the community level through the health extension workers and health facilities. Usually, LLINs are distributed by periodic mass campaigns that occur about every 3 years in a rotation basis [2].

Beyond mere emphasis on overall coverage, malaria prevention services in general and LLIN ownership and IRS status in particular, should be fair regardless of socioeconomic status over time. Both LLIN and IRS are mainly financed through the MoH either from donation or direct government budgeting. Therefore, unarguably, the benefits from these publicly financed interventions shall be distributed equitably. A test regarding this normative position is that the odds of malaria infection should be the same for all socioeconomic classes [18]. Worrall et al. [19], based on review of several literatures, and Filmer [20], using 29 Demographic and Heath Surveys (DHS) data from 22 countries, establish a very weak link between malaria incidence and wealth status at micro-level. No differences were found at the household level in the incidence of fever between the poor and less poor [20]. Similarly, a recent study by Gari et al. from the same area also found no significant association between wealth status and incidence of malaria [7].

The underlying assumption is that at individual or household level, the odds of malarial infection is quite similar if either of them are not using the preventive measures. Therefore the argument that the socioeconomically better-off are in a better position to access the other non-publicly financed means of malaria prevention including mosquito repellent or window meshes could not be justified given that the availability in rural setting is limited. For this reason, this paper emphasizes that malaria prevention interventions (LLINs and IRS) should be owned equitably at any given time. However, the Ethiopian government has committed to follow pro-poor universal health service delivery strategy, which goes beyond policy statements of creating equal access to health services for all groups of population [21].

In a nutshell, in this malaria elimination and eradication era, information on the equity dimension is more important than ever for priority setting and resource allocation [22–24]. In contrast, little is currently known about who benefits from prevention efforts. Where are those freely distributed bed nets? Who owns them? Whose houses are sprayed or not? These questions reflect concerns about social justice and fairness, and have so far not systematically been investigated. In this paper, household survey data were used to evaluate the socioeconomic related dimension of inequalities in malaria prevention interventions (LLIN and IRS) in a district in south-central Ethiopia. Therefore, the hypothesis is that the poor families are equally likely to own the LLINs and to live in a house treated with IRS compared with better-offs.

## Methods

### Study area and participants

This study is part of a large cluster randomized controlled trial, which aim to evaluate the combined use of LLINs and IRS against each intervention alone in preventing malarial infection [25]. This study uses data from a baseline household survey conducted in July–August 2014 in Adami Tullu district of Oromia region in south-central Ethiopia. The survey was conducted in 13 villages, located within 5 km from the shore of Lake Ziway. Overall, 31,284 individuals from 6069 households were included.

The district is situated in the heart of the Great Rift Valley. Most of the villages are located in the lowland portion, while the elevation ranges from low altitude of 1500 m to higher altitude of 2300 m above sea level. The area is partly dry and arid, where malaria is largely seasonal, and partly swampy and marshy, where malaria is largely perennial.

### Data collection

The data were obtained from the head of the household by trained nurses who performed face-to-face interviews using a pre-tested structured questionnaire. The questionnaire contains information about socioeconomic position, including questions about demographic situation, ownership of different household assets, ownership and utilization of malaria prevention services, and general health service utilization.

### Data analysis and model specifications

#### Measuring socioeconomic status

The two recommended ways to consider for measuring socioeconomic status is to use consumption expenditure levels of the households and to use asset based wealth index. Nonetheless, consumption expenditure measurement in the present situation would have been likely to be unreliable, since most people base their livelihood on subsistence farming for own consumption, so that the market value of much of the produced is never realized [26]. For this reason, principal components analysis (PCA) was used to construct a wealth index based on household characteristics: such as, availability of various household assets, housing conditions, water source, and type of latrine facility. An equation provided by Filmer and Pritchett [27] was used to calculate the wealth index (*A*), for individual *i,* defined as follows:

$$A_{i} = \mathop \sum \limits_{k} \left[ {f_{k} \frac{{(a_{ik} - \bar{a}_{k} )}}{{s_{k} }}} \right]$$

where, *a*_*ik*_ is the value of household characteristics or *k* for household *i* (i.e. 0 = if the household didn’t own that specific characteristics; 1 = if the household own that characteristics), $\bar{a}$_*k*_ is the sample mean, *s*_*k*_ is the sample standard deviation, and fk are the weights (eigenvectors) extracted from the first principal component which are correlation matrix of the data [26, 27].

### Measuring LLIN ownership and IRS status

The primary health outcome variables are household level LLIN ownership and IRS status. LLIN ownership and IRS with insecticide were defined as “the household owns at least one functional LLIN” and “the house is sprayed within the last 12 months”, respectively. LLIN ownership was measured by direct observation by the data collectors while IRS status was assessed based on what the household head reported. A binary logit regression model was employed in order to predict the probability of LLIN ownership and IRS status of the households. The unit of analysis in this study is at household level.

### Measuring inequality

The main measures of inequality is the concentration curve and concentration index (CI) [28]. The concentration curve plots the cumulative percentage of the health variable (LLIN and IRS ownership) on the y-axis against the cumulative percentage of the population on x-axis, ranked by wealth index beginning with the poorest, and ending with the least poor (richest). If everyone irrespective of the wealth status has exactly the same value of the prevention measures, then the concentration curve will be a straight diagonal line, from the bottom left corner to the top right corner. Besides visual inspection of the concentration curve, a dominance test using the multiple comparison approach was applied to examine for statistical significance of the difference between the concentration curve and the line of equality (diagonal).

A concentration index is a relative measure of inequality. A CI ranges from –1 to 1, with a value of 0 indicating perfect equity. The index takes a negative value when the variable of interest is concentrated among the poorest groups and a positive value when it is concentrated among the richest group [28, 29]. The conventional concentration index (CI) is a covariance between LLIN ownership/IRS treatment (*y*_*i*_) and the socioeconomic rank (*R*_*i*_) of that household, multiplied by two, and then the whole expression divided by the mean of the outcome variable (μ).

$$CI({\text{y}}) = \frac{{2 * \text{cov} \left( {y_{i} , R_{i} } \right)}}{\upmu }$$

However, the health outcome variables (LLIN ownership and IRS) were binary in which case a normalized concentration index is preferred over the conventional CI. “Erreygers normalized concentration index” was employed, which is provided by Erreygers and Van Ourti [30] as follows;

$$CCI = 4*\mu *CI(y)$$

where, $CI\left( y \right)$ is the generalized concentration index and *μ* is the mean (in this case proportion of LLIN ownership or IRS coverage).

### Decomposition analysis

Wagstaff et al. proved that concentration index are decomposable into its contributing factors [31]. They showed that, for each factor, its contribution is the product of the sensitivity of the outcome variable with respect to that factor and the degree of socioeconomic status inequality in that factor. They provide a linear additive regression model for outcome variable y, against to a set of k determinants, $x_{k}$, as follows:

$${\text{y}} = \alpha + \mathop \sum \limits_{k} B_{k} x_{k } + \varepsilon$$

Then concentration index for y (i.e. Concentration index of LLIN) (CIy) can be written as:

$${\text{CIy}} = \mathop \sum \limits_{k} \left( {\frac{{B_{k} \bar{x}_{k} }}{\mu }} \right)C_{k } + \left( {\frac{{GC_{\varepsilon } }}{\mu }} \right)$$

where $\bar{x}_{k}$ is the mean value of the determinant $x_{k }$, μ is the mean of the outcome variables (LLIN), $C_{k }$ is the concentration index of the determinant $x_{k }$; *GCε* is the residual component that captures wealth-related inequality in LLIN that is not accounted for by systematic variation in determinants across wealth groups, and $\left( {\frac{{{\text{B}}_{\text{k}} \bar{\text{x}}_{\text{k}} }}{\upmu }} \right)$ is the impact of each determinant on the probability of LLIN ownership and represents the elasticity (*η*_*k*_) of the outcome variable with respect to the determinant $x_{k}$ evaluated at the mean *y*. In this paper, this decomposition technique was used to estimate, and compare the contribution of socioeconomic effects to that of education, religion, ethnicity, household size, place of residence (village), housing conditions, access to infrastructure (electricity and piped water), ownership and access to mass-media and telecommunication service (radio, television, mobile telephone). All analyses were conducted using STATA version 14 [32].

## Results

### Characteristics of the study population

Table 1 shows a summary of the study participants and distribution of LLINs and IRS among households classified into different socioeconomic and demographic groups. A total of 6069 households were enrolled into the study. The mean household size was 5.1 (range from 1 to 14). The majority of the study participants were Oromo (5512, 91 %), muslim (5199, 86 %) and illiterate (3335, 55 %).

**Table 1:** Description of malaria prevention by different household characteristics

Household characteristics	N (%)	LLIN n (%)	IRS n (%)	Both LLIN and IRS n (%)	Nothing at all n (%)
Ethnicity					
Oromo	5512 (90.82)	640 (11.61)	4034 (73.19)	497 (9.02)	1335 (24.22)
Amhara	46 (0.76)	5(10.87)	28 (60.87)	5 (10.87)	18 (39.13)
Gurage	58 (0.96)	8 (13.79)	47 (81.03)	7 (12.07)	10 (17.24)
Other ethnicity	453 (7.48)	51 (11.23)	290 (64.02)	48 (10.60)	160 (35.32)
Religion					
Muslim	5199 (85.66)	562 (10.81)	3739 (71.92)	436 (8.39)	1334 (25.66)
Orthodox christian	709 (11.68)	128 (18.05)	557 (78.56)	112 (15.80)	136 (19.18)
Protestant christian	149 (2.46)	12 (8.05)	96 (64.43)	8 (5.37)	49 (32.89)
Other religion^a^	12 (0.20)	2 (16.67)	7 (58.33)	1 (8.33)	4 (33.33)
Educational status					
Illiterate	3336 (54.95)	334 (10.01)	2584 (77.48)	278 (8.34)	695 (20.84)
Can read and write only	562 (9.26)	67 (11.92)	421 (74.91)	49 (8.72)	123 (21.89)
Elementary (1–4)	519 (8.55)	102 (19.65)	342 (65.90)	78 (15.03)	153 (29.48)
Junior Elementary (5–8)	972 (16.02)	120 (12.33)	636 (65.36)	90 (9.25)	307 (31.55)
High school (9–12)	513 (8.45)	71 (13.84)	344 (67.06)	52 (10.14)	150 (29.24)
Above high school	77 (1.30)	9 (11.69)	52 (67.53)	9 (11.69)	25 (32.47)
NR^b^	90 (1.47)	1 (1.11)	20 (22.22)	1 (1.11)	70 (77.78)
Wealth quintiles					
Poorest	1214 (20.00)	98 (8.07)	882 (72.65)	77 (6.34)	311 (25.62)
2nd poorest	1214 (20.00)	112 (9.23)	912 (75.12)	80 (6.59)	270 (22.24)
Middle	1214 (20.00)	144 (11.86)	916 (75.45)	124 (10.21)	278 (22.90)
2nd richest	1213 (20.00)	172 (14.18)	851 (70.16)	138 (11.38)	328 (27.04)
Richest	1214 (20.00)	178 (14.66)	838 (69.03)	138 (11.37)	336 (27.68)
Overall total	6069 (100.00)	704 (11.60)	4399 (72.48)	557 (9.18)	1523 (25.09)

^a^Other religion practiced in that area was *Wakefeta*

^b^No response (missing) for educational status question

### LLIN ownership and IRS coverage

The overall LLIN ownership was 704 (11.6 %), ranging from 98 (8.0 %) in the poorest quintile to 178 (14.7 %) in the richest quintile. Regarding IRS, about three quarters of the houses were sprayed in the last 12 months. A quarter of households had neither own any LLIN nor their house was sprayed, whereas 557 (9.2 %) of the households owned LLIN meanwhile their house is sprayed in the last 12 months.

The binary logit model for LLIN ownership show that households wealth status, larger household size, having a latrine, and having a radio were significantly positively associated with LLIN ownership, where as having a separate cooking space from the main room and having a larger number of sleeping spaces, were significantly and negatively associate with household LLIN ownership (Table 2). Similarly, the logit model for the IRS shows that educational status of head of the household was significantly associated with the probability of having IRS (Table 3).

**Table 2:** Logit model predicting the probability of LLIN ownership

Variable	Coef.	Robust SE	P value	[95 % Conf. interval]	
Wealth status (ref. = reachest Q)					
Poorest Q	−0.8390	0.2541	0.001	−1.3370	−0.3410
Second poorest Q	−0.6149	0.2152	0.004	−1.0367	−0.1931
Middle Q	−0.3240	0.1748	0.064	−0.6666	0.0187
Second richest	−0.1249	0.1501	0.405	−0.4190	0.1693
Ethnicity (ref. = other ethnicity)					
Oromo	−0.4490	0.2419	0.063	−0.9232	0.0251
Amhara	−0.3835	0.5992	0.522	−1.5580	0.7909
Gurage	0.6176	0.4832	0.201	−0.3294	1.5646
Religion (ref. = other religion)					
Orthodox	0.3698	0.7026	0.599	−1.0072	1.7468
Muslim	0.1984	0.7006	0.777	−1.1747	1.5715
Protestant	−0.1818	0.7495	0.808	−1.6508	1.2873
Education (ref. = above high school)					
Illiterate	0.3061	0.3550	0.388	−0.3896	1.0019
Can read and write only	0.4762	0.3534	0.178	−0.2163	1.1688
Elementary (1 − 4)	0.7490	0.3633	0.039	0.0369	1.4612
Junior Elementary (5 − 8)	0.3550	0.3786	0.348	−0.3871	1.0971
High School (9 − 12)	0.5916	0.3955	0.135	−0.1835	1.3667
Household size	0.0556	0.0261	0.033	0.0044	0.1068
Villages (ref. = Kebele #13)					
Kebele1	2.3848	0.4098	0.000	1.5815	3.1881
Kebele2	4.7088	0.4908	0.000	3.7468	5.6707
Kebele3	1.5564	0.3727	0.000	0.8260	2.2868
Kebele4	1.9868	0.3083	0.000	1.3826	2.5910
Kebele5	0.5895	0.4206	0.161	−0.2348	1.4137
Kebele6	−0.5098	0.8708	0.558	−2.2165	1.1968
Kebele7	0.7979	0.5923	0.178	−0.3629	1.9587
Kebele8	−1.6184	1.0035	0.107	−3.5852	0.3484
Kebele9	−0.5220	0.4334	0.228	−1.3714	0.3275
Kebele10	1.6388	0.3679	0.000	0.9176	2.3600
Kebele11	0.9948	0.4361	0.023	0.1402	1.8495
Kebele12	1.7502	0.3978	0.000	0.9705	2.5299
Housing					
Has a bed	0.0791	0.1433	0.581	−0.2018	0.3599
Has a separate cooking space	−0.3018	0.1148	0.009	−0.5269	−0.0768
Number of living rooms	0.0579	0.1010	0.566	−0.1400	0.2558
Number of sleeping space	−0.2148	0.0769	0.005	−0.3656	−0.0641
Has a latrine	0.3659	0.1145	0.001	0.1414	0.5904
Roof (1 corrugated iron, 0 thatch/leaf)	−0.2038	0.1322	0.123	−0.4629	0.0553
Wall(1 mud &wood and better, 0 rudimentary)	0.2154	0.3255	0.508	−0.4226	0.8535
Communication access					
Has television	0.2903	0.1897	0.126	−0.0814	0.6620
Has radio	0.2446	0.0995	0.014	0.0495	0.4397
Has mobile telephone	0.0059	0.1294	0.964	−0.2478	0.2595
Infrastructure and utility					
Has electricity	0.0295	0.1829	0.872	−0.3290	0.3880
Use piped water for drinking	0.0191	0.1695	0.910	−0.3132	0.3514
_Constant	−3.6198	0.9835	0.000	−5.5475	−1.6921

**Table 3:** Logit model predicting the probability of IRS status of the household

Variable	Coef.	Robust SE	P value	[95 % Conf. interval]	
Wealth status (ref. = reachest Q)					
Poorest Q	−0.7766	0.3046	0.0110	−1.3737	−0.1795
Second poorest Q	−0.6166	0.2203	0.0050	−1.0483	−0.1849
Middle Q	−0.4146	0.1786	0.0200	−0.7647	−0.0645
Second richest	−0.4790	0.1380	0.0010	−0.7495	−0.2085
Ethnicity (ref. = other ethnicity)					
Oromo	−0.5795	0.1772	0.0010	−0.9269	−0.2322
Amhara	−0.5909	0.3829	0.1230	−1.3414	0.1596
Gurage	0.8654	0.3414	0.0110	0.1963	1.5345
Religion (ref. = other religion)					
Orthodox	0.2692	0.5776	0.6410	−0.8629	1.4013
Muslim	0.0498	0.5325	0.9250	−0.9938	1.0935
Protestant	−0.0749	0.5819	0.8980	−1.2154	1.0656
Education (ref. = above high school)					
Illiterate	1.2899	0.3798	0.0010	0.5454	2.0344
Can read and write only	1.1435	0.3658	0.0020	0.4265	1.8605
Elementary (1−4)	1.1823	0.4136	0.0040	0.3717	1.9929
Junior elementary (5−8)	1.1041	0.3995	0.0060	0.3210	1.8872
High school (9−12)	1.0489	0.4004	0.0090	0.2641	1.8336
Household size	0.0331	0.0195	0.0890	−0.0050	0.0712
Villages (ref. = Kebele #13)					
Kebele1	−0.1902	0.4541	0.6750	−1.0802	0.6998
Kebele2	−1.8311	0.5471	0.0010	−2.9035	−0.7588
Kebele3	0.8239	0.4677	0.0780	−0.0927	1.7405
Kebele4	2.9881	0.6129	0.0000	1.7869	4.1894
Kebele5	−0.9067	0.3628	0.0120	−1.6178	−0.1956
Kebele6	−0.6001	0.8312	0.4700	−2.2291	1.0289
Kebele7	2.3747	0.4985	0.0000	1.3977	3.3516
Kebele8	−6.1193	0.8757	0.0000	−7.8357	−4.4030
Kebele9	−2.1782	0.4496	0.0000	−3.0595	−1.2970
Kebele10	−0.3213	0.4660	0.4910	−1.2347	0.5921
Kebele11	1.0652	0.4392	0.0150	0.2044	1.9260
Kebele12	−0.0950	0.4770	0.8420	−1.0300	0.8400
Housing					
Has a bed	0.2100	0.1189	0.0770	−0.0231	0.4431
Has a separate cooking space	−0.0274	0.1247	0.8260	−0.2718	0.2169
Number of living rooms	−0.0380	0.1217	0.7550	−0.2764	0.2005
Number of sleeping space	−0.0574	0.0949	0.5450	−0.2434	0.1287
Has a latrine	−0.3592	0.1077	0.0010	−0.5703	−0.1481
Roof (1 corrugated iron, 0 thatch/leaf)	−0.3412	0.1275	0.0070	−0.5912	−0.0913
Wall (1 mud and wood and better, 0 rudimentary)	−0.2931	0.2303	0.2030	−0.7445	0.1583
Communication access					
Has television	0.0543	0.2082	0.7940	−0.3538	0.4624
Has radio	0.1525	0.1001	0.1270	−0.0436	0.3487
Has mobile telephone	0.0499	0.0990	0.6140	−0.1441	0.2440
Infrastructure and utility					
Has electricity	−0.5746	0.1847	0.0020	−0.9366	−0.2126
Use piped water for drinking	−0.6268	0.2007	0.0020	−1.0201	−0.2335
_Constant	2.0583	0.8134	0.0110	0.4642	3.6525

### Equity in LLIN and IRS ownership

**Fig. 1:**
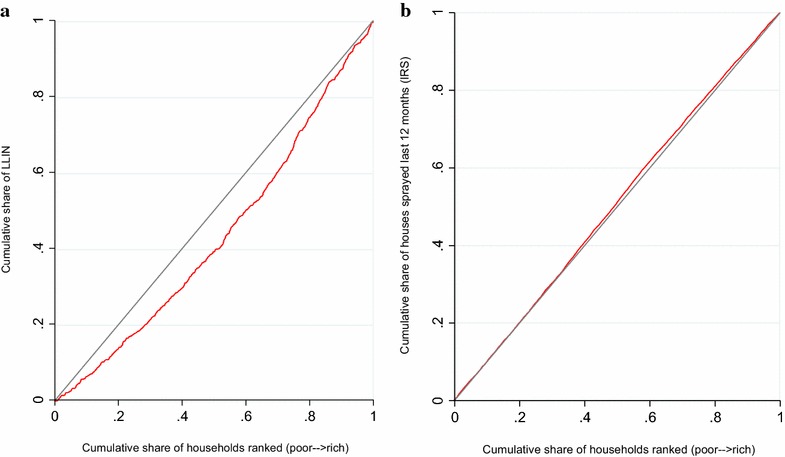
Concentrations curves for LLIN ownership (**a**), IRS in the last 12 months (**b**)

The concentration curve for LLIN is clearly below the diagonal line (Fig. 1a), indicating a pro-rich distribution. The dominance test based on the multiple comparison approach indicates that the concentration curve is significantly below the line of equality at 19 evenly spaced points. Similarly, the Erreygers normalized concentration index of 0.06270 (SE = 0.03898) was significantly different from zero (P < 0.0001) (Table 4).

**Table 4:** Erreygers normalised and generalized concentration indices for LLIN and IRS distribution

Concentration Index (CI)	Malaria prevention programs	
	LLIN	IRS
Erreygers normalised CI	0.06270**	−0.03834
Generalized CI	0.13495**	−0.01323
95 % confidence interval	(0.09526, 0.17465)**	(−0.02232, −0.00413)*
Standard error (delta method)	0.03898	0.01139

Significant at 0.001**, 0.01* level of significance

On the other hand, the concentration curve for IRS is closely aligned with the diagonal line (Fig. 1b), indicating that there was no noticeable difference in houses sprayed according to different socioeconomic status. The Erreygers normalized concentration index of −0.03834 (SE = 0.01139) for the IRS was not significantly different from zero.

The decomposition analysis shows that inequality in ownership of LLIN is largely driven by the wealth itself (90.77 %), whereas ethnicity (4.25 %), religion (2.63 %) and educational status (3.4 %) of the head of the household had little influence on inequality. Difference in housing situation, access to mass media and telecommunication, and household size, were also found to be predominantly contributing for the inequality. The positive or negative sign of the CI or the percentage contribution in Table 5 demonstrates that the factor was concentrated among rich or poor household respectively. For example, higher educational attainment, larger household size, those who have bed and latrine, as reported in Table 5, are concentrated among the richest households. The percentage contribution of wealth is an estimate of the pure effect of wealth on the total inequality, adjusting for other relevant factors.

**Table 5:** Decomposition of Erreygers normalised concentration index for LLIN ownership in Adami Tullu, Ethiopia, 2014

Variable	Concentration index (CI)	Contribution to CI	Percentage contribution (%)
Wealth status		0.0569	90.77
Ethnicity (1 other ethnicity, 0 otherwise)		−0.0027	−4.26
Religion (1 other religion, 0 otherwise)		0.0016	2.63
Educational status of the head of household		0.0021	3.40
Household size	0.1047	0.0094	14.94
Village (1 village 13, 0 otherwise)		0.0015	2.37
Housing situation		−*0.0264*	**−** *42.19*
Has a bed	0.1996	0.0022	3.54
Has a separate cooking space	0.3253	−0.0157	−24.98
Number of living rooms	0.0804	−0.0001	−0.20
Number of sleeping space	0.1090	−0.0128	−20.40
Has a latrine	0.1758	0.0103	16.45
Roof (1 corrugated iron, 0 thatch/leaf)	0.2808	−0.0103	−16.35
Wall (1 mud and wood and better, 0 rudimentary)	−0.0028	−0.0002	−0.25
Access to mass media and communication		*0.0156*	*24.93*
Has television	0.7805	0.0053	8.48
Has radio	0.3656	0.0106	16.88
Has mobile telephone	0.2422	−0.0003	−0.43
Infrastructure and utility		0.0011	*1.74*
Has electricity	0.2367	0.0009	1.36
Use piped water for drinking	0.1081	0.0002	0.38
Residual		*0.00356*	0.00
Total	*0.0627*		

Subtotal are highlighted in italics

## Discussion

This study is the first to provide empirical evidence about socioeconomic inequalities in malaria prevention interventions from a district in Ethiopia. This study tries to evaluate the household level coverage and equity dimension of LLIN ownership and IRS status. The main finding from this study indicates very low ownership of LLIN and low coverage of IRS in general, while the findings on the coverage across wealth status were mixed. On one side, LLINs were distributed significantly in favor of the rich, while IRS on the other side was distributed equitably regardless of household wealth status.

The very low ownership of LLIN (11.6 %) found in this study is totally unparalleled with finding from most of other studies [33, 34] including the malaria indicator survey [16]. The reason for this big difference might be due to the gap in the time period between this survey and the last LLIN distribution conducted in the area. A report from the district indicates that the last LLINs distribution, for most of the villages, was conducted 2 years ago by the Districts’ Health Office. Nonetheless, the national malaria prevention guidelines dictates that all sleeping spaces in malaria endemic areas should be covered at least with one LLIN at any time [2].

The observed significant difference in both LLINs ownership and IRS status across villages might be mainly due to the districts’ malaria prevention schedule which is conducted on a rotating basis. Those villages which receive the interventions recently reported higher ownership while others received a couple of years back report low.

In the bivariate analysis, the associations between LLIN ownership and having separate cooking space or having more number of sleeping space were non-significant. However, in the multiple logit model (i.e. adjusted for wealth status, cluster, ethnicity, religion, education, and household size), both “having a separate cooking space” and “more number of sleeping space” are significantly negatively associated with LLIN ownership, which is contrary to prior expectations. The first speculation is that households with limited number of sleeping space for hanging the nets might apply them less frequently and subsequently the nets might have survived longer, while nets in household which had adequate space for hanging-up worn-out quicker. Loha et al. also reports that lack of convenient space was a barrier for hang-up the bed nets from quite similar sociodemographic area [35]. These finding have important implications that the national LLINs distribution programme should critically consider number of sleeping space in addition to household size based allotment of the nets for optimizing the efficiency of available LLINs. Moreover, the relationship between LLIN ownership, number of sleeping spaces and useful life time of the LLIN is not sufficiently well understood, which warrants more research.

LLINs are significantly more likely to be owned by the rich, even when analyses are adjusted for village. In a situation where the coverage is low and the inequality in ownership is high, an empirical study [36] and a mathematical model [37] highlight that community wide protection of the LLINs could be diminished. The uses of LLINs decrease probability of bites of mosquitoes for the ultimate users without significantly decreasing the population of mosquito. Consequently, the potential advantage of the ‘positive externality’ to those who could not own by themselves might be nullified.

Various studies from sub-Saharan Africa consistently report the cost as a main barrier to ownership of LLIN among the poorest households [38]. In this study area, LLINs were distributed free of charge, and the cost argument is, therefore, less apparent. Several questions need consideration to better understand the causalities. From the demand side—one may ask whether the poor are reluctant to collect their share from the health posts? Are the poor unable to avail themselves on the dates and place of distribution? Did the LLINs in the poorest households wear out faster and got lost because of improper handling? Do the poor sell the LLINs received? The current study didn’t investigate these matters. However, in the field site stay, the authors frequently observed that several of LLINs were used for other purposes, such as collecting crops and vegetables in the farm, for fencing or as a fishing net). As a consequence, it could be that the “useful life” of the LLINs differs between the socio-economic strata.

In contrast, the equitable distribution of the IRS between socio-economic strata is surely a notable achievement and might be partly driven by the nature of the intervention, which requires minimal compliance from the household side. The spray is conducted using community-based approaches, including annual campaigns, administered from the District Health Office. The IRS programme has been well accepted and implemented for more than half a century throughout the country [39]. Thus, this coverage mainly reflects the performance of the health system and the IRS is a dependable vector control option.

Based on the decomposition analysis, the wealth status was the single most dominant factor for the overall socioeconomic related inequality in LLIN ownership. This finding suggests that any effort in improving the welfare of the household should be considered as a fight against malaria and vice versa [10]. Housing condition and access to mass media and telecommunication also contributed to the observed inequality. This finding has an implication that inequality in LLINs ownership is partly driven by differential access to sources of information. The government needs to consider the LLIN promotion strategies targeting the poor. These findings have important policy implications that sole emphasis on the distribution of LLINs is not sufficient to ensure neither the coverage nor the equity; it should be accompanied by teaching how to properly handle and effectively use the LLINs. In order to achieve equity in ownership of LLIN throughout the year, a priority, in both scale-up and replacement distribution should be given to the poor.

There is an ongoing debate on which specific concentration index is the most appropriate based on the properties of the indices and the nature of the variable under investigation. However, there seems to be increasing support that the concentration index needs to be adjusted for the binary nature of health outcome variables. This study apply Errygers normalized concentration index and its decomposition—appropriate measures of inequality for binary outcome [30].

These findings should be interpreted carefully, especially the wealth measurement and the classification method employed was applicable for relative ranking only. In a rural situation where more than a quarter of the total population is living in absolute poverty [40], even those households in the middle or second richest quintile could be below poverty line by standardized living status measurement. The other concern could be raised about the generalizability of the findings. The proportion of the population who owned a LLIN was much lower than comparable studies, and this study was conducted in a single district. This study may not be a full representative of the malaria situation of a rural Ethiopia.

A third limitation to this study is that it only focuses on horizontal equity. Socioeconomic related inequalities in health services are only considered unfair, when they do not correspond to differences in need for health care across socioeconomic groups. In other way, horizontal equity means that households in equal need for the service should receive equal service irrespective of other characteristics such as wealth status, ethnicity, religion or geographical location. On the other side, vertical equity describes the extent to which households with greater needs received more service [29]. For example:, households which are located more close to the mosquito breeding site might have higher LLIN need while this study did not consider standardization based on difference in need.

## Conclusion

The ownership of LLIN is significantly pro-rich, while IRS status is equitable across socio-economic strata. The distribution campaign should be followed by periodic refill based on continuous monitoring data. Local data on ‘useful life’ of LLIN and tracking information should be ready for timely planning of LLIN distribution.

## Abbreviations

CI: concentration index
DALY: disability adjusted life year
DHS: demographic and heath survey
ICER: incremental cost effectiveness ratio
IRB: institutional review board
IRB: institutional review board
IRS: indoor residual spraying
ITN: insecticide treated net
ITN: insecticide treated net
LLIN: long-lasting insecticidal net
MIS: malaria indicator survey
MoH: Ministry of Health
PCA: principal components analysis
SE: standard error
WHO: World Health Organization
